# Dynamic mode decomposition of inertial particle caustics in Taylor–Green flow

**DOI:** 10.1038/s41598-021-89953-3

**Published:** 2021-05-17

**Authors:** Omstavan Samant, Jaya Kumar Alageshan, Sarveshwar Sharma, Animesh Kuley

**Affiliations:** 1 Centre for Fusion, Space and Astrophysics, University of Warwick, Coventry, CV4 7AL UK; 2 Department of Physics, Indian Institute of Science, Bangalore, 560012 India; 3 Institute For Plasma Research, Gandhinagar, Gujarat 382428 India

**Keywords:** Physics, Fluid dynamics, Computational science

## Abstract

Inertial particles advected by a background flow can show complex structures. We consider inertial particles in a 2D Taylor–Green (TG) flow and characterize particle dynamics as a function of the particle’s Stokes number using dynamic mode decomposition (DMD) method from particle image velocimetry (PIV) like-data. We observe the formation of caustic structures and analyze them using DMD to (a) determine the Stokes number of the particles, and (b) estimate the particle Stokes number composition. Our analysis in this idealized flow will provide useful insight to analyze inertial particles in more complex or turbulent flows. We propose that the DMD technique can be used to perform similar analysis on an experimental system.

## Introduction

Advection of particles, such as dust or aerosol by a background flow is a ubiquitous phenomena. And the study of dispersion of these inertial particles are of immense interest both for applied and natural processes, in particular, to analyze oil spills in oceans^1–4^, dispersion of pollutants and toxic elements^5,6^, suspended particles in aquatic systems^7^, formation of clouds^8,9^ and volcanic plumes^10^, and the effect of the flow patterns generated by the breathing action and cough on the dispersion of the aerosol particles are crucial to understand the spread of COVID-19 virus^11–16^. Numerical studies of inertial particle dispersion in different types of flows, ranging from static^17^ to turbulent^18,19^ flows have shown that the particles display complex dynamical behaviours like formation of fractal clusters^18^ and caustics^20^. The analysis of the structures formed by the particles encode information about the Stokes number of the particles and the flow patterns.

Experimental techniques such as particle image velocimetry (PIV) have been used to track particles and extract velocity profiles^21^ when it is possible to identify individual particles in an image, but not with certainty to track it between images. If the particle concentration is so low that it is possible to follow an individual particle it is called particle tracking velocimetry (PTV). While similar techniques have been adopted to track particles in simulations, the averaging process reduces the spatial resolution, which is critical in our application. In simulations, the Osiptov’s method^22^ have been used to extract caustic features^20^, which track each particle in PTV like situations but fail for PIV like data. We propose the use dynamical mode decomposition (DMD) based scheme to obtain the spatio-temporal particle distributions as a representative for particle density. DMD methods have been used to extract coherent structures in simulations and experiments^23–26^ of fluids. We use the DMD method to analyze and extract the features of the caustics to (a) determine the Stokes number of the particles, and (b) estimate the relative particle concentrations in a bi-disperse Stokes number system. Our approach can also be extended to multiple Stokes number poly-disperse systems.

This paper is organized as follows. In “Model” we present the form of the TG flow, minimal model of an inertial particle, and the relevant numerical simulation details. In “Observations” we show the formation of caustic structures and analyze them using DMD method in “Analysis” and demonstrate how we extract the caustic wavefronts from the DMD mode. We use the position and the gradient of the wavefront in the DMD eigen mode to estimate the Stokes number and the composition of a bi-disperse Stokes number systems in “Determination of Stokes number from DMD” and “Estimation of particle concentration” respectively, and present our conclusions in “Conclusions”.

## Model

We consider a 2D lattice of vortices in the form of a Taylor–Green (TG) flow. The TG flow is a steady state solution to the forced, incompressible Navier–Stokes equation and can be considered a convection model in 2D^27,28^. Such a flow can be experimentally setup using ion solutions in an array of magnets^29^. The TG flow is given by the vorticity field as

1 $$\begin{aligned} \omega (x,y) = \omega _0 \; \sin \left( \frac{2\pi x}{L}\right) \; \sin \left( \frac{2\pi y}{L}\right) \end{aligned}$$

and the corresponding velocity field is

2 $$\begin{aligned} \mathbf {u}(x,y) = V_0 \begin{bmatrix} &{} \sin \left( \frac{2\pi x}{L}\right) \; \cos \left( \frac{2\pi y}{L}\right) \\ -&{} \cos \left( \frac{2\pi x}{L}\right) \; \sin \left( \frac{2\pi y}{L}\right) \end{bmatrix} \end{aligned}$$

where $x,y \in [0,L)$ and are periodic, $\mathbf {u}$ is the Eulerian velocity field such that $\omega = \nabla \times \mathbf {u}$, with $\omega _0 = 4\pi V_0/L$. We choose $V_0$ as the velocity scale and *L* as the length scale and write the system parameters in corresponding dimensionless form. We model the aerosol particles as small rigid spheres, which are effectively points, that have density different from the surrounding fluid. The equation of motion of the inertial particles in a background flow given by the simplified Maxey-Riley approximation^17^ for small particles that are much denser than the fluid are

3 $$\begin{aligned} \frac{d\mathbf {x}}{dt}= & {} \mathbf {v} \nonumber \\ \frac{d\mathbf {v}}{dt}= & {} \frac{1}{St} \left( \mathbf {u}-\mathbf {v}\right) \end{aligned}$$

where *St* is the Stokes number which captures the effect of particle inertia, $\mathbf {x}$ is the particle position and $\mathbf {v}$ is the particle velocity (see Appendix A for validity of the equations). The case when $St\rightarrow 0$ the particles act as tracers that follow the velocity stream lines and the Eq. (3) leads to $\mathbf {v}=\mathbf {u}$. We use RK4 numerical scheme to discretize and evolve the particle positions and velocities. Furthermore, we use periodic boundary conditions, such that the particles are reintroduced into the system when they exit the boundary. In our analysis we set the time step to $\Delta t = 0.01 \left( L/V_0\right)$.

## Results

### Observations

**Figure 1:**
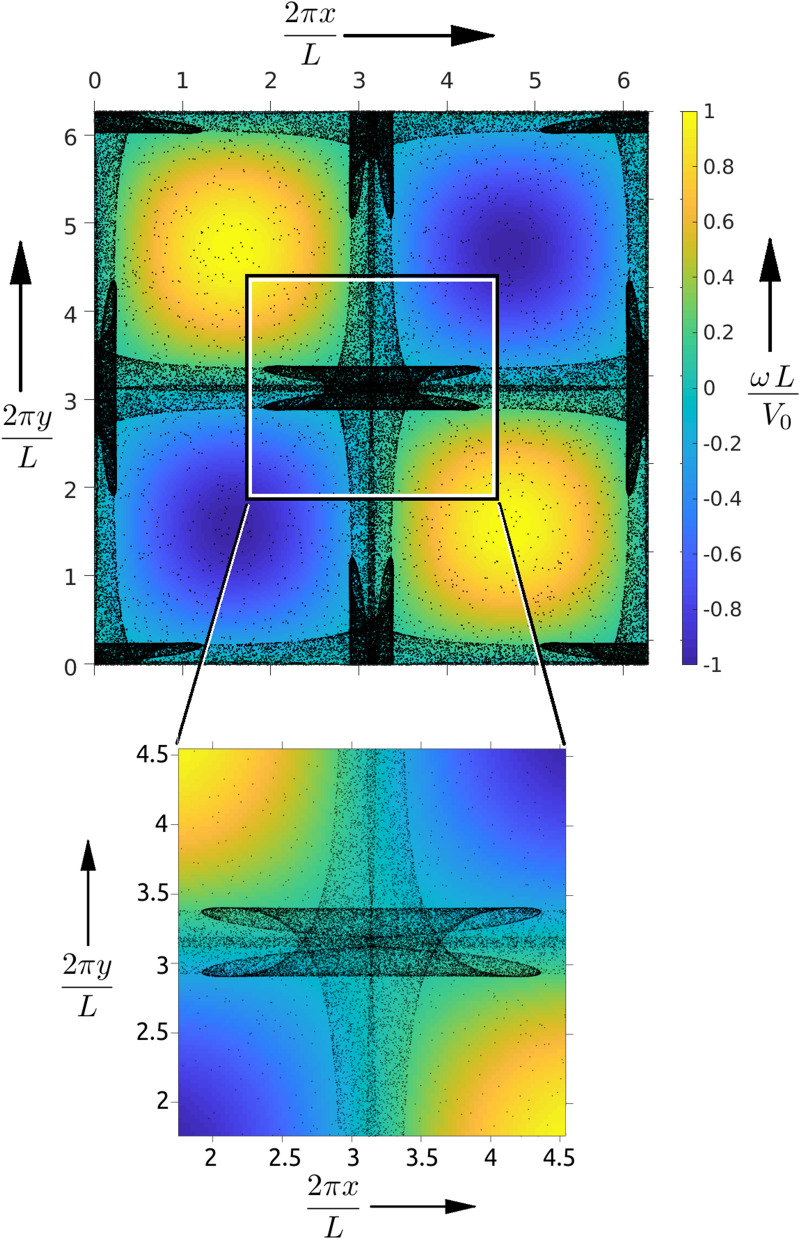
A snapshot of the 2D spatial particle distribution in **(a)** at $t=750 \, \Delta t$ shows the complex structure formed by the mono-dispersed inertial particles with $St=1.0$ in a periodic domain of size $L\times L$. The background color-map corresponds to the vorticity field (with values given by the colorbar on the left side of the top panel) and the particles are plotted using black markers. Notice that the particles in the high vorticity regions have moved out towards the regions bounding the vortices. The zoomed-in version in **(b)** shows the details of the caustic structures around the central region of the domain. See the video C.1 for the evolution of the caustic structures. The figures were generated using Matlab 2020a, https://www.mathworks.com/products/matlab.html.

A typical feature of inertial particles in a background flow is that they are expelled from high vorticity regions. In a background flow with vortices, the inertial particles can form caustics^20^. So in the case of TG flow, the inertial particles tend to accumulate along the low vorticity regions that separate the vortices. We simulate the dynamics of inertial particles in a TG flow and observe spatial regions where the Lagrangian velocity field is multi-valued, which are referred to as caustics^30–32^ (see Fig. 3a).

Figure 1 shows the caustic structure formed by a mono-disperse inertial particle system, when we start the simulation with the particle positions initialized with uniform random distribution within the $L\times L$ box, and setting the initial velocities of the particles to zero. In the long time limit, the particles accumulate along a curve^33^. We observe that the caustics that form in the transient state are robust and stable to small perturbations (see Appendix A), whereas the steady state structures break up and lead to chaos^33^. Furthermore, the steady state behaviour strongly depends on the system size and the boundary conditions.

We find that for a range of Stokes numbers the caustic structures preserve their shapes; and their sizes depend on *St*. In the following section we use dynamic mode decomposition (DMD) to extract features of the structures, in particular the caustic wavefront, and study its size dependence on the Stokes number. Furthermore, the sharpness of the caustic wavefront enables us to detect and extract their sizes even in presence of poly-disperse Stokes number systems.

### Analysis

The caustics in Fig. 1 have a complex structure and in the presence of multiple Stokes number particles resolving these structures from a single snap-shot is hard. Therefore we employ the spatio-temporal data in the form of a video sequence that contains $\mathscr {F}$ frames of $N\times N$ pixel images and analyze them using the dynamic mode decomposition (DMD) method.

DMD is a data analysis technique that has been used to extract coherent structures in fluid dynamic systems^34^, where it is able to extract different modes that are similar to normal modes in linear dynamical systems. The DMD is a data-driven technique introduced by Schmid as a numerical procedure for extracting dynamical features from flow data^24^. The DMD algorithm takes in a time-series data in the form of vectors $\{\mathbf {v}_1,\mathbf {v}_2,...\mathbf {v}_T\}$ and estimates a linear dynamical system that can generate a map

4 $$\begin{aligned} \mathbf {v}_{i+1} = \mathscr {A} \; \mathbf {v}_i \end{aligned}$$

where $\mathscr {A}$ is an $N^2 \times N^2$ matrix and the eigenvectors of $\mathscr {A}$ form the DMD modes, with the corresponding eigenvalues. Finally, the eigenvectors are reshaped into $N\times N$ pixel image to obtain the modes. A Singular Value Decomposition (SVD) based algorithm for estimating the DMD modes is described in Appendix B.

**Figure 2:**
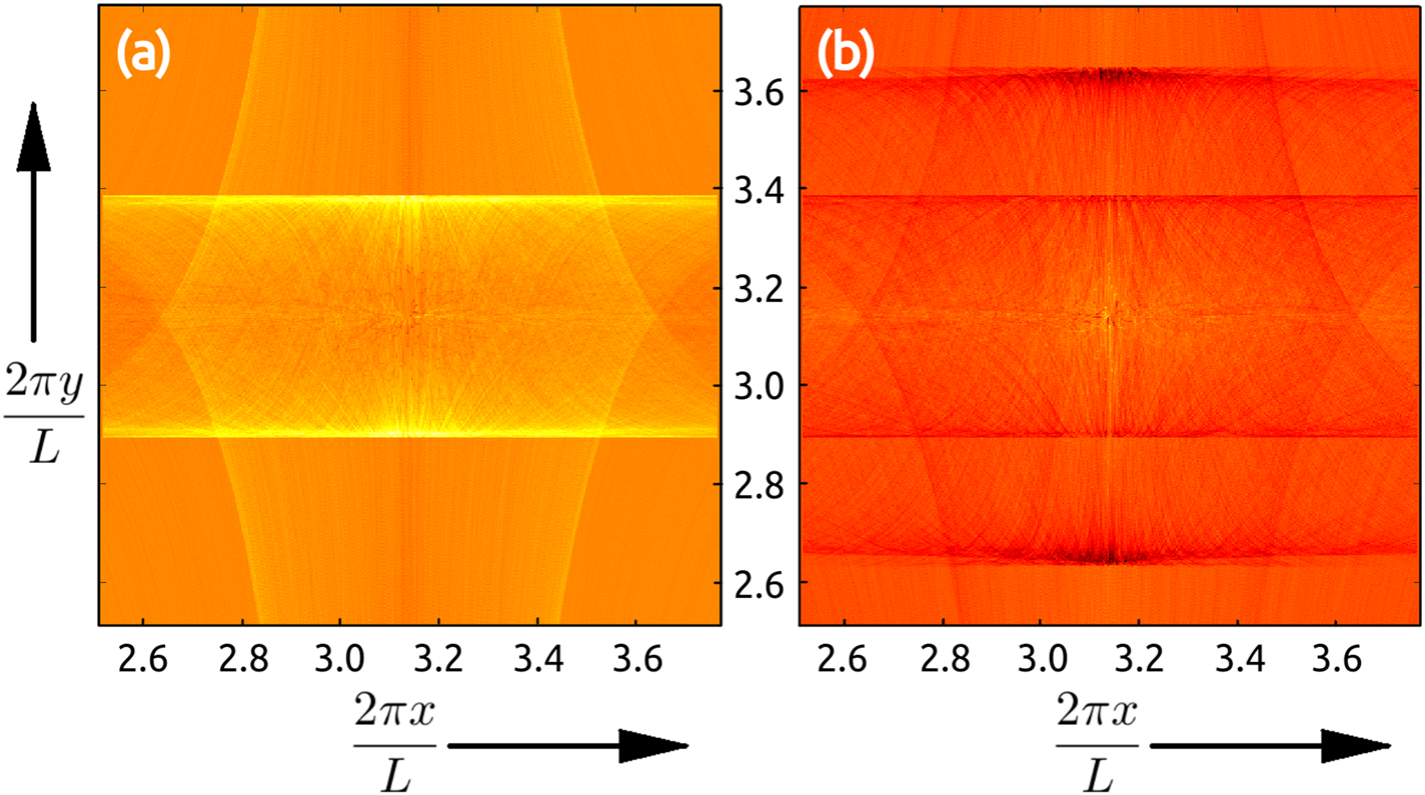
The highest singular DMD eigenvector, $\mathscr {D}^{(1)}$, obtained for: **(a)** $St=1.0$ mono-disperse system, **(b)** a bi-disperse mixture of $St=\{1, 2\}$ with 7:3 ratio of initial particle concentrations. The horizontal lines correspond to the caustic wavefronts, and the number of such fronts indicate the different Stokes number particles. Also notice that the wavefronts corresponding to $St=1.0$ appear around the same values of *y* in both **(a,b)**, indicating that the positions of DMD wavefronts are not perturbed by the presence of particles with different Stokes numbers. The figures were created using Matlab 2020a, https://www.mathworks.com/products/matlab.html.

**Figure 3:**
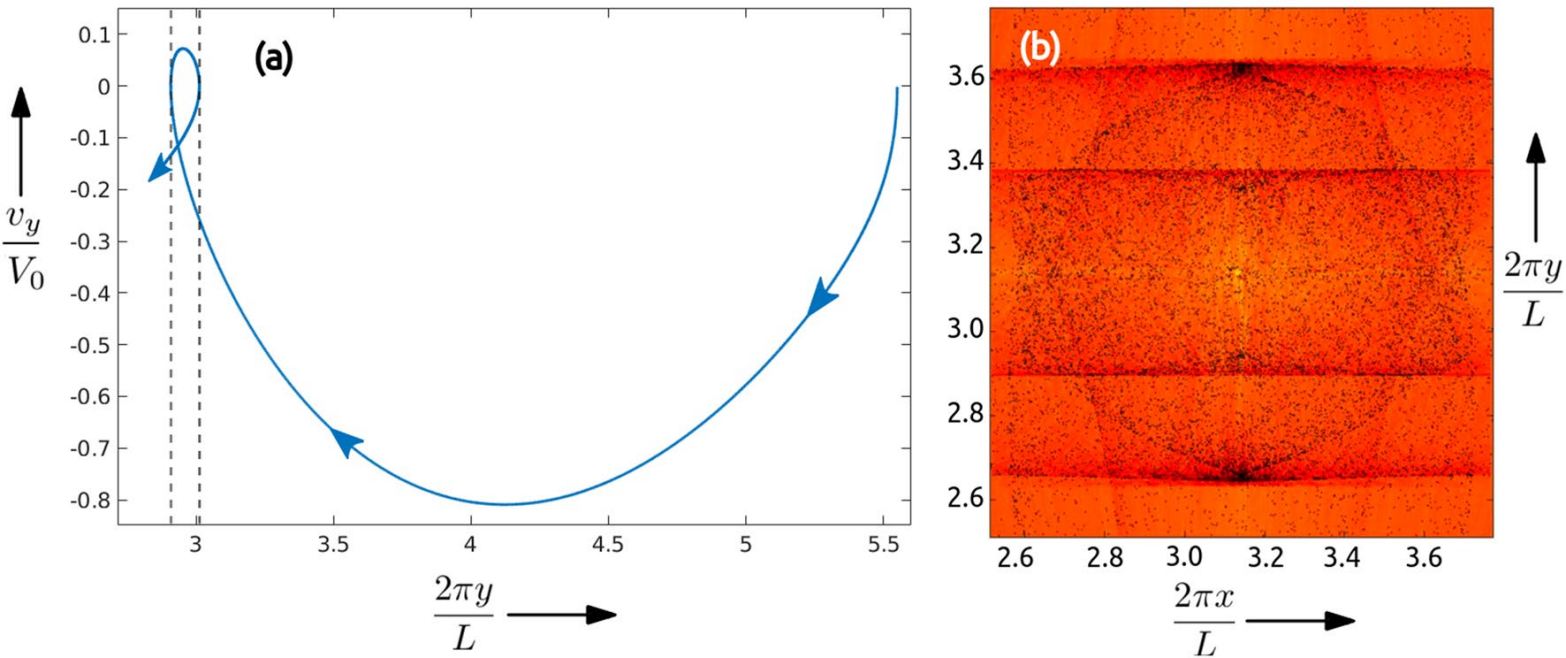
**(a)** The particle trajectory in the reduced phase space, $(y,v_y)$, shows the multi-valued nature of the casutics in velocity between the dotted vertical lines^32^. The movie in C.2 shows the evolution of the bi-disperse particles overlaid on the corresponding DMD from Fig. 2b and the plot (b) shows a snap-shot at the $650{\rm th}$ iteration step when the caustics and the DMD wavefronts coincide. Notice that the first DMD picks out only the slow moving horizontal caustics. The plot and the figure was generated using Matlab 2020a, https://www.mathworks.com/products/matlab.html.

Let *i* stand for the iteration number such that the particles are in their stationary initial state and start their evolution at $i=0$. Then for our data, the vectors $v_i$ are obtained by rearranging the $N\times N$ pixel images at instant *i* into $N^2\times 1$ vector. In our DMD analysis we employ $\{v_{250}, v_{251},...v_{750}\}$ (i.e. $\mathscr {F}=500$), as the modes obtained are the sharpest for this range. Let $\mathscr {D}^{(\alpha )}(j,k)$ represent the $(j,k){\rm th}$ pixel of the $\alpha {\rm th}$ eigenmode, ordered in terms of decreasing absolute eigenvalue. Since the caustics are localized around the central region of the domain, we use a zoomed-in region of size $512 \times 512$ pixels (i.e. $N=512$) in our analysis, as shown in Fig. 1. We find that the highest singular eigenvalue mode, namely $\mathscr {D}^{(1)}$, shown in Fig. 2a highlights a straight-line caustic structure, which we refer to as the wavefront. The eigenvalues of other DMD modes decay exponentially. We observe that the position of the wavefront in $\mathscr {D}^{(1)}$ has a systematic dependence on the Stokes number, and to extract this relation we detect the location of the wavefronts using edge detection techniques. Similarly, when we perform DMD analysis on a bi-disperse system $\mathscr {D}^{(1)}(j,k)$ shows two distinct wavefronts (see Fig. 2b) corresponding to the two different Stokes numbers; and here DMD uses the velocity information to unambiguously extract the wave fronts. In particular, Fig. 3a shows the reduced phase space portrait of a typical particle which form the caustic^32^ and Fig. 3b shows the particles overlaid on top of the DMD that demonstrates the DMD’s ability to extract the caustic structures. Furthermore, we find that the intensity of each wavefront compared to the background, which we refer to as prominence^35^, depends on the corresponding initial particle concentrations in the system.

We now prescribe a method to extract the position of the wavefront from DMD. We use a Sobel operator^36^ such that the vertical gradient of the first DMD mode is given by

5 $$\begin{aligned} G(j,k) \; = \; \frac{\partial \mathscr {D}^{(1)}(j,k)}{\partial y} \; \approx \; \frac{1}{\Delta y} \begin{bmatrix} \;\;1 &{} \;\;2 &{} \;\;1 \\ \;\;0 &{} \;\;0 &{} \;\;0 \\ -1 &{} -2 &{} -1 \end{bmatrix} \circledast \; \mathscr {D}^{(1)}(j,k) \end{aligned}$$

where $\circledast$ represents the 2D convolution operator^37^, and $\Delta y$ is the spacing in DMD along the *y*-axis. We then sum over the values in the x-direction to get a 1D function of y as

6 $$\begin{aligned} \langle G \rangle _x = \int _0^L G(j,k)\; dx \;\; \approx \; \sum _{j=1}^{N} G(j,k) \; \Delta x \end{aligned}$$

where $\Delta x$ is the spacing in DMD along the *x*-axis, and we choose a square grid with $\Delta x = \Delta y$ such that $\langle G \rangle _x$ is by definition independent of the grid spacing and is dimensionless. In the next section we describe how the location and the value of the peaks in $\langle G \rangle _x$ can be used to find the Stokes number of the particles and the relative initial concentrations in the case of a bi-disperse system.

#### Determination of Stokes number from DMD

**Figure 4:**
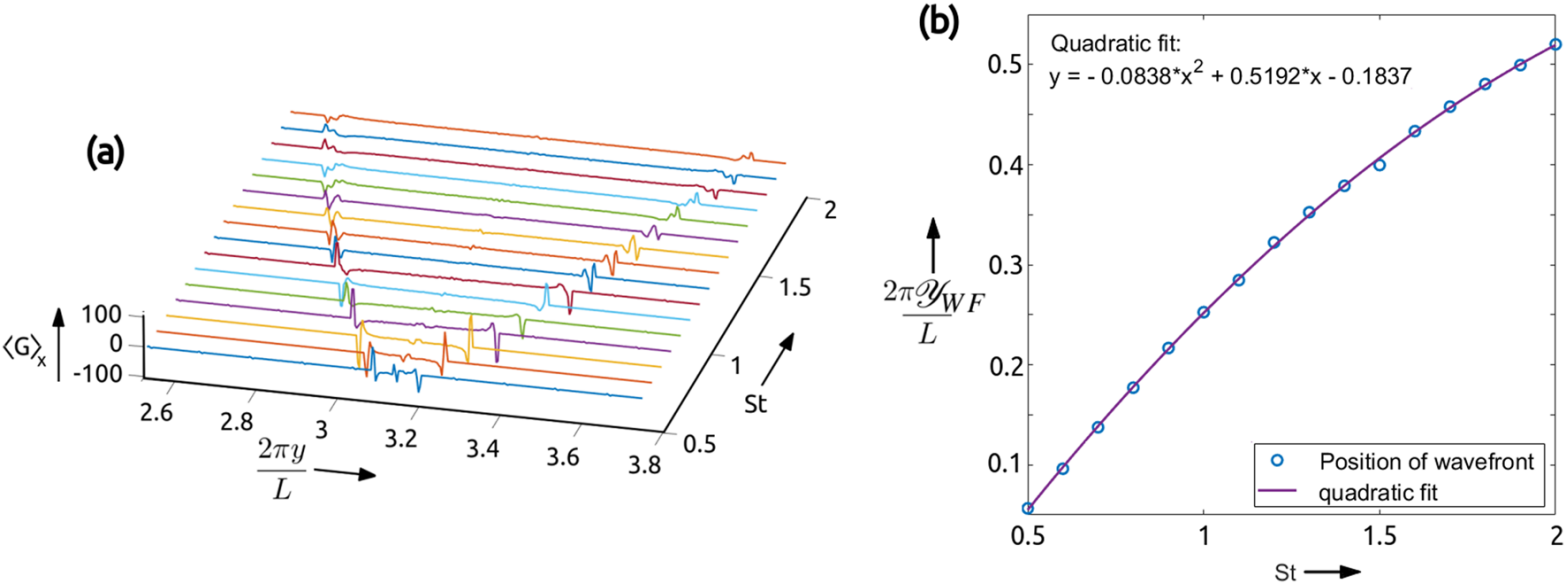
In **(a)** we show the plots of $\langle G \rangle _x$ obtained for different values of the Stokes number from mono-disperse systems. We extract the location of the peaks in $\langle G \rangle _x$ for each *St*, as defined in Eq. (7), to generate the plot in **(b)** and we find that the $\mathscr {Y}_{WF}$ and *St* have a quadratic dependence. The plots were generated using Matlab 2020a, https://www.mathworks.com/products/matlab.html.

The $\langle G \rangle _x$ is obtained from the DMD as described in “Analysis” by simulating mono-disperse Stokes number particle systems to generate the plots in Fig. 4a which shows $\langle G \rangle _x$ as a function of *St*. The alignment of the peaks in $\langle G \rangle _x$ along a curve indicates a systematic dependence of the location of wavefront on the Stokes number. To extract this relation we first get the location of the caustic wavefront from the domain center using the position of the peaks in $\langle G \rangle _x$ given by

7 $$\begin{aligned} \mathscr {Y}_{WF} = \arg \max _y\langle G \rangle _x - \frac{L}{2} \end{aligned}$$

where $\arg \max _y$ gives the value of *y* for which $\langle G \rangle _x$ is maximum. We then plot $\mathscr {Y}_{WF}$ as a function of *St* as shown in Fig. 4b. Using a non-linear least squares fit method we find that the relation is of the form $\mathscr {Y}_{WF} \sim a \; St^2 + b \; St + c$, with values of the parameters *a*, *b*, and *c* as indicated in Fig. 4b, where *x* represents *St*. Now, extrapolating the fit we find that $\mathscr {Y}_{WF}=0$ at $St=0.3767$ and becomes multi-valued for $St>3.0979$, thus setting the limits on the validity of the relation. In stagnation-point flows below a certain value of Stokes number normally there are no caustics, as shown by Healy et al.^22^; the lower cut-off for the Stokes number in our case could be indicative of a similar phenomenon. The fact that St = 0.37 appears as a hard cut-off for caustics could be circumstantial and tied to the choice of initial condition and flows can actually display caustics at any Stokes number. Furthermore, we restrict our study to $St < 3$ motivated by similar studies in literature^33^. As described in Cencini et al.^38^, the Maxey–Riley approximation in Eq. (3) is not valid for Stokes numbers greater than 3 as the back-reaction of the particle on the flow becomes important. The three parameters in the relation can be estimated experimentally using calibrated measurements and the relation can be used to predict the *St* of new particle systems. In particular we demonstrate that the above method can be generalized to work in case of bi-disperse system.

**Figure 5:**
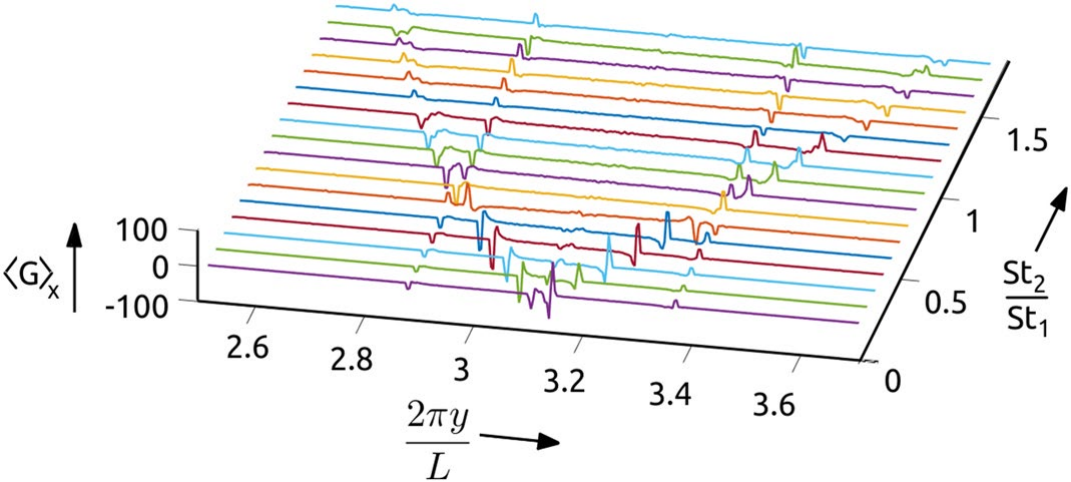
Plots of $\langle G \rangle _x$ for a bi-disperse system as a function of *y* and the ratio of the the two particle Stokes numbers, with one of the Stokes number fixed at one ($St_1=1.0$), and varying $St_2$. Notice that the peaks in $\langle G \rangle _x$ corresponding to $St_1$ are aligned at the same location along *y*, whereas the peaks due to $St_2$ show similar trend as the plots for mono-disperse systems in Fig. 4a. This plot was generated in Matlab 2020a, https://www.mathworks.com/products/matlab.html.

As shown in Fig. 2b, for a bi-disperse system the DMD has two sets of caustic wavefronts, corresponding to each Stokes number. Now we set one of the Stokes number fixed at one ($St_1 = 1.0$), vary the $St_2$ and find $\langle G \rangle _x$ to generate the plots in Fig. 5. The results in Fig. 5 show that even in the bi-disperse system the caustic wavefront has the same characteristic behaviour on the Stokes number as the mono-disperse system. In particular, the wavefront corresponding to $St_1$ has a fixed location and the wavefront due to $St_2$ preserves the dependence on $\mathscr {Y}_{WF}$ of the mono-disperse system. Our studies with poly-disperse *St* systems show that the caustic wavefronts can be used to find the Stokes number of different particles in the system using the relation obtained from a mono-disperse system.

#### Estimation of particle concentration

**Figure 6:**
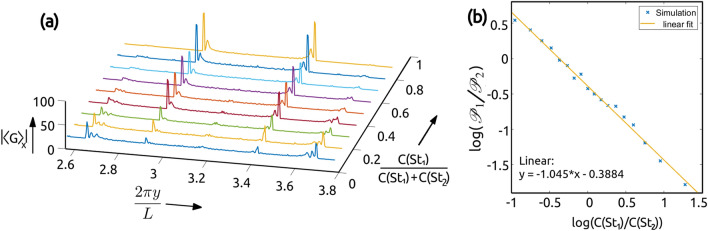
The plot **(a)** shows the variation in the absolute value of $\langle G \rangle _x$ for a bi-disperse system, with $St_1=1$ and $St_2=2$ particles, for various initial concentration fraction $C(St_1)/(C(St_1)+C(St_2))$. Notice that the peaks corresponding to each wavefront is not unique and have a finite spread in y. In **(b)** the relation between the prominence corresponding to $St_1$ and $St_2$ are given by $\mathscr {P}_1$, $\mathscr {P}_2$ respectively, as a function of the initial particle concentrations *C* is shown in a log–log plot. The linear fit shows that the ratios of the peaks of $\hat{|\langle G \rangle |}$ and the ratios of the concentration are related by a power-law, with a power close to −1. The plots were generated using Matlab 2020a, https://www.mathworks.com/products/matlab.html.

Until now the bi-disperse systems that we considered had equal number of $St_1$ and $St_2$ particles, with uniform initial distribution in space. Now we study the variation in $\mathscr {D}^{(1)}$ w.r.t. the change in relative number of particles. We observe that the intensity of the wavefront or the gradient in the DMD image depends on the number of particles or the initial uniform concentration, denoted by *C*(*St*).

The variable $\langle G \rangle _x$ gives the gradient of $\mathscr {D}^{(1)}$ along the vertical direction and the magnitude of the gradient indicates sharpness of the wavefronts (see Fig. 6a). To measure the sharpness of the wavefront we define a ”prominence” parameter, $\mathscr {P}$, as the sum of the non zero values of $\bar{|\langle G \rangle _x|}$ in the neighbourhood of the wavefront, which takes into account multiple peaks in the vicinity of the wavefront. We find that the prominence of the wavefront has a systematic dependence on the initial concentration of the corresponding Stokes number particles and from Fig. 6b we find that on a log–log plot the relation is linear, with a slope approximately equal to −1. This implies that in a bi-disperse system the ratio of the prominence is inversely related to the ratio of initial concentrations. We can use this relation to predict the concentration of various Stokes number particles in a system.

## Conclusions

We study the dynamics of inertial particles in a Taylor–Green flow with periodic boundary conditions in 2D. In a minimal model of inertial particles we observe that for a mono-disperse Stokes number system, starting from a uniform distribution of stationary particles, the particle distribution forms caustics in the strain dominated region of the flow. We use the DMD method to analyze the PIV-like time-series data of the spatio-temporal particle distribution and find that the largest absolute eigenvalue mode is effective in extracting the caustic wavefront-like structure. We notice that (a) the position of the wavefront depends on the particle Stokes number and employ standard image processing techniques to quantitatively extract a quadratic relation. Using this relation we can predict the Stokes number from the wavefront position. Furthermore, we find that for a bi-disperse system the DMD is able to extract two different wavefronts corresponding to each Stokes number and the positions of each wavefront follow the same quadratic relation as in the case of mono-disperse system. We also observe that (b) the sharpness of the wavefront in the DMD, measured in terms of prominence, depends on the initial particle concentration and find that for a bi-disperse Stokes number system the ratio of the wavefront prominence is inversely proportional to the corresponding Stokes number initial concentration. Hence the measurement of prominence can be used to estimate the concentration of the corresponding Stokes number particles.

We propose that the DMD technique can be used to analyze real experimental PIV data of caustics and perform similar analysis to extract information about the Stokes numbers and concentrations of the particles. In future we will consider detailed Navier-Stokes equation for the self-consistent evolution of the velocities and analyze the caustic structures in 3D^39^ and turbulent flows^18,40^. Contrary to the Taylor-Green flow case, a single dominant DMD mode does not capture the complete features of the caustics in a turbulent flow. As described in Marensi et al.^41^, symmetries play an important role in data driven modal expansions for turbulent flows and would entail the use of symmetry-reduced dynamic mode decomposition (SRDMD).

### Supplementary Information

Supplementary Legends.

Supplementary Video 1.

Supplementary Video 2.

## A Validity of particle dynamics

The particle dynamics we use in our study is a special case of^17^

8 $$\begin{aligned} \frac{d\mathbf {x}}{dt}= & {} \mathbf {v} \nonumber \\ \frac{d\mathbf {v}}{dt}= & {} \frac{1}{St} \left( \mathbf {u}-\mathbf {v}\right) + R \; \left( \mathbf {u}.\nabla \mathbf {u} + \frac{1}{2} \mathbf {v}.\nabla \mathbf {u} \right) + \mathbf {\eta }(\mathbf {v},t) \end{aligned}$$

where $\eta$ is the white noise and $R=\rho _f/(\rho _p+\rho _f/2)$ for density of particle $\rho _p$ and density of fluid $\rho _f$. We present our results for $\eta =0$ and $R=0$, and use small values of these parameters to verify the stability of our results. In Wang et al.^33^ the authors show that for $R=0$ the system is not chaotic. It will be interesting to check for $R>0$ if the DMD modes can capture the transition of the system from periodic to chaotic states.

## B DMD algorithm

The $N\times N$ pixel image at $k{\rm th}$ instant, $I^k(i,j)$, is rearranged into an $N^2\times 1$ vector $X^k(m)$ (note that we subtract the mean value from $X^k(m)$). Now the $\mathscr {F}$ sequence of image frames are appended together to form $N^2 \times N_T$ matrix *Y*. Let the $N^2 \times (\mathscr {F}-1)$ dimensional matrix formed from the first $(\mathscr {F}-1)$ frames be $Y_1$ and the last $(\mathscr {F}-1)$ frames be $Y_2$, i.e.

9 $$\begin{aligned} \left[ I^k \right] _{N\times N}\longrightarrow & {} \left[ X^k\right] _{N^2 \times 1} \nonumber \\ \left[ X^1 | X^2 | ... | X^{N_T} \right]\longrightarrow & {} \left[ Y \right] _{N^2 \times N_T} \nonumber \\ \left[ X^1 | X^2 | ... | X^{(N_T-1)} \right]\longrightarrow & {} \left[ Y_1 \right] _{N^2 \times (N_T-1)} \nonumber \\ \left[ X^2 | X^3 | ... | X^{N_T} \right]\longrightarrow & {} \left[ Y_2 \right] _{N^2 \times (N_T-1)} \end{aligned}$$

We find the singular value decomposition (SVD) of the matrix $Y_1$, such that $Y_1 = U\Sigma V^*$, where *U* is a $N^2\times N^2$ complex unitary matrix, $\Sigma$ is an $N^2 \times (\mathscr {F}-1)$ rectangular diagonal matrix with non-negative real numbers on the diagonal, and *V* is a $(\mathscr {F}-1)\times (\mathscr {F}-1)$ real or complex unitary matrix.

Now choose a lower dimensional SVD matrices made up of first $n_T (<<\mathscr {F})$ columns, represented by $\tilde{U}$, $\tilde{V}$, and the first $n_T\times n_T$ block of $\Sigma$ as $\tilde{\Sigma }$. Define a matrix $n_T\times n_T$ matrix *A* as

10 $$\begin{aligned} A = \tilde{U}^* \; Y_2 \; \tilde{V} \; \tilde{\Sigma }^{-1} \end{aligned}$$

Then the dynamic modes are the $N^2 \times 1$ eigenvectors of matrix *A* that is rearranged into $N\times N$ matrix.

## C Movies

The movies referred to in the text are available in the additional materials.

### C.1

See ”Vid1.mp4”.

### C.2

See ”Vid2.mp4”.
