# Supplementary material for: Dynamic mode decomposition of inertial particle caustics in Taylor–Green flow
Source: Sci Rep. 2021 May 17;11:10456. doi: 10.1038/s41598-021-89953-3 (PMC8128860; doi:10.1038/s41598-021-89953-3)
Supplement: Supplementary file 1 — Supplementary Legends. [file 41598_2021_89953_MOESM1_ESM.pdf]

Supplementary material

Dynamic Mode Decomposition of inertial particle caustics in Taylor-Green flow

Omstavan Samant[1], Jaya Kumar Alageshan[2,*], Sarveshwar Sharma[3], and Animesh Kuley[2]
[1] Centre for Fusion, Space and Astrophysics, University of Warwick, Coventry, CV4 7AL, UK.
[2] Department of Physics, Indian Institute of Science, Bangalore, India - 560012.
[3] Institute For Plasma Research, Gandhinagar, Gujarat, India - 382428.
[*] *jkumar.res@gmail.com*

The supplemental material contains two videos. The caption corresponding to each of the videos are given below:

C.1: Vid1.mp4

Caption: The video shows the evolution of St=1 inertial particles in a Taylor-Green flow. The colormap in the background represents the strength of the vorticity field. Figure. 1 in the manuscript shows a snapshot of the particle distribution at t = 750 Δt.

C.2: Vid2.mp4

Caption: To demonstrate the correlation between the particle caustics and the DMD, we overlay the evolution of particles on top of the DMD obtained for a bi-disperse system. Notice that at 650th iteration the caustic front and the DMD fronts match.
